# Raman Spectroscopy and Machine Learning for IDH Genotyping of Unprocessed Glioma Biopsies

**DOI:** 10.3390/cancers13164196

**Published:** 2021-08-20

**Authors:** Tommaso Sciortino, Riccardo Secoli, Ester d’Amico, Sara Moccia, Marco Conti Nibali, Lorenzo Gay, Marco Rossi, Nicolò Pecco, Antonella Castellano, Elena De Momi, Bethania Fernandes, Marco Riva, Lorenzo Bello

**Affiliations:** 1 Unit of Oncological Neurosurgery, Humanitas Clinical and Research Center—IRCCS, 20089 Rozzano, Italy; tommaso.sciortino@unimi.it (T.S.); marco.conti@unimi.it (M.C.N.); lorenzo.gay@unimi.it (L.G.); marco.rossi2@unimi.it (M.R.); lorenzo.bello@unimi.it (L.B.); 2 Department of Oncology and Hemato-Oncology, Università degli Studi di Milano, 20122 Milano, Italy; 3 The Hamlyn Centre for Robotic Surgery, Institute of Global Health Innovation, Imperial College London, Exhibition Road, London SW7 2AZ, UK; r.secoli@imperial.ac.uk; 4 Department of Electronics, Information and Bioengineering, Politecnico di Milano, Piazza Leonardo da Vinci 32, 20133 Milano, Italy; ester.damico@mail.polimi.it (E.D.); elena.demomi@polimi.it (E.D.M.); 5 The BioRobotics Institute and Department of Excellence in Robotics and AI, Scuola Superiore Sant’Anna, 56127 Pisa, Italy; sara.moccia@santannapisa.it; 6 Department of Neuroradiology, IRCCS Ospedale San Raffaele, 20132 Milan, Italy; pecco.nicolo@hsr.it; 7 Neuroradiology Unit, IRCCS San Raffaele and Vita-Salute San Raffaele University, 20132 Milan, Italy; castellano.antonella@hsr.it; 8 Unit of Pathology, Humanitas Clinical and Research Center—IRCCS, Via Manzoni 56, 20089 Rozzano, Italy; bethania.fernandes@humanitas.it; 9 Department of Medical Biotechnology and Translational Medicine, Università degli Studi di Milano, 20122 Milan, Italy

**Keywords:** raman spectroscopy, neuro-oncology, classification, glioma, machine learning, isocitrate dehydrogenase (IDH)

## Abstract

**Simple Summary:**

Isocitrate dehydrogenase (IDH) mutation is one of the most important prognostic markers in glioma tumors. Raman spectroscopy (RS) is an optical technique with great potential in intraoperative molecular diagnosis and surgical guidance. We analyzed RS’s ability to detect the IDH mutation onto unprocessed glioma biopsies. A total of 2073 Raman spectra were extracted from 38 tumor specimens. From the 103 Raman shifts screened, we identified 52 shifts (related to lipids, collagen, DNA and cholesterol/phospholipids) with the highest performance in the distinction of the two groups. We described 18 shifts never used before for IDH detection with RS in fresh or frozen samples. We were able to distinguish between IDH-mutated and IDH-wild-type tumors with an accuracy and precision of 87%. RS showed optimal accuracy and precision in discriminating IDH-mutated glioma from IDH-wild-type tumors ex-vivo onto fresh surgical specimens.

**Abstract:**

Isocitrate dehydrogenase (IDH) mutational status is pivotal in the management of gliomas. Patients with IDH-mutated (IDH-MUT) tumors have a better prognosis and benefit more from extended surgical resection than IDH wild-type (IDH-WT). Raman spectroscopy (RS) is a minimally invasive optical technique with great potential for intraoperative diagnosis. We evaluated the RS’s ability to characterize the IDH mutational status onto unprocessed glioma biopsies. We extracted 2073 Raman spectra from thirty-eight unprocessed samples. The classification performance was assessed using the eXtreme Gradient Boosted trees (XGB) and Support Vector Machine with Radial Basis Function kernel (RBF-SVM). Measured Raman spectra displayed differences between IDH-MUT and IDH-WT tumor tissue. From the 103 Raman shifts screened as input features, the cross-validation loop identified 52 shifts with the highest performance in the distinction of the two groups. Raman analysis showed differences in spectral features of lipids, collagen, DNA and cholesterol/phospholipids. We were able to distinguish between IDH-MUT and IDH-WT tumors with an accuracy and precision of 87%. RS is a valuable and accurate tool for characterizing the mutational status of IDH mutation in unprocessed glioma samples. This study improves RS knowledge for future personalized surgical strategy or in situ target therapies for glioma tumors.

## 1. Introduction

Molecular classification of gliomas, the most common primary malignant brain tumors in adults, allows a better prognostic and therapeutic stratification and, to date, is the standard evaluation [1]. The treatment involves, when feasible, surgical resection [2] followed, eventually, by radiotherapy and chemotherapy. Both molecular and immunohistochemical data are routinely determined several days after samples collection, foreclosing a personalized intraoperative surgical and oncological strategy.

The Isocitrate dehydrogenase (IDH) gene mutation is one of the most critical molecular markers to influence oncological outcomes and tumor response to adjuvant treatments in low and high-grade gliomas. Patients with IDH-mutated (IDH-MUT) tumors have better overall and progression-free survival and benefit from more extended surgical resection than IDH wild-type (IDH-WT) glioma patients. IDH mutation modifies the metabolic activity and catabolic production of tumoral cells dramatically. In IDH mutant cells, the conversion of isocitrate to α-ketoglutarate (α-KG) is abolished, whereas the production of D-2-hydroxyglutarate (D-2-HG) is enhanced. D-2-HG, an oncometabolite, has a primary role in gliomas oncogenesis, altering different processes involved in DNA and histones methylation and gene expression that drives the cell toward a staminal phenotype. 

The extent of surgical resection is one of the strongest prognostic element in IDH- MUT glioma management [2,3,4]. Surgical excision has to be pursued up to functional boundaries (i.e., eloquent cortical and subcortical sites) to achieve a gross-total or a supra-total resection, possibly resulting in a transient postoperative neurological impairment that can delay or, in the worst scenarios, exclude adjuvant therapies. Therefore, the balance between immediate postoperative patients’ functional integrity and maximal tumor resection should consider the tumor’s biological behaviour. In this light, non-invasive devices supplying fast molecular analysis are advocated.

Raman spectroscopy (RS) [5,6] and similar optical technology (e.g., Fourier Transform Infrared Spectroscopy (FTIR) [7,8]) resulted effective tools to discriminate between cancer and normal tissue and, more recently, to investigate IDH mutational status [9,10]. RS studies using fresh tissue samples are of primary importance to improve Raman measurement in vivo, avoiding the well-known samples artefacts due to the histological blocks processing and storage [11]. Only few studies can provide RS data from fresh glioma tissue [10]. 

In this study, we investigated RS’s capacity to distinguish IDH-MUT glioma biopsies from IDH-WT glioma tumor ex-vivo on fresh tissue samples.

## 2. Materials and Methods

### 2.1. Study Population and Experimental Design

Thirty-eight (38) tumor samples were collected from adult subjects undergoing surgery for presumptive glioma tumors. All procedures were performed with imaging and neurophysiological guidance [12,13] in order to achieve, when feasible, a safe supra-marginal resection [3]. We collected 21 samples from IDH-mutated tumors and 17 samples from IDH-WT tumors. All patients signed informed consent for the procedure. The study was conducted in line with the 1964 Declaration of Helsinki and later amendments and authorized by the ethic committee. Demographic, clinical, and spectroscopic features were registered, analyzed and were reported in Table 1. 

### 2.2. Samples Collection and Analysis

All patients were submitted to surgery for presumptive low- or high-grade glioma. Magnetic resonance imaging was performed 24 h before surgery and employed as input for the Neuronavigation system (Curve, Brainlab. A.G., Munich, Germany). During the surgical procedure, a small part of the bulk tumor tissue was collected, with accurate imaging verification of the sampling area. The samples were immediately provided to the Raman analyst for processing, cleaned with NaCl solution, and placed under a CaF_2_ window before Raman investigation.

**Figure 1:**
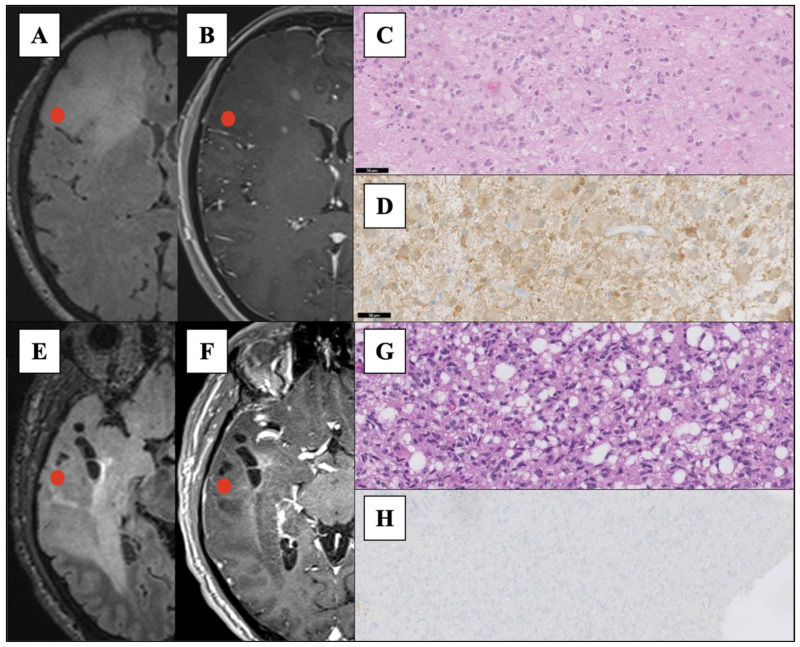
(**A**,**B**): Preoperative MRI ((**A**) axial fluid-attenuated inversion recovery image and (**B**) T1 post-gadolinium axial scan) of a right frontal tumor consistent with Anaplastic Astrocytoma IDH-1 mutant, grade III (WHO 2016 [1]) after histological and molecular analysis. The red spots indicate the surgical location of the specimen labelled as IDH-MUT. (**C**,**D**) Histological evaluation of the sample analyzed after Raman analysis: panel (**C**) shows Hematoxylin-and eosin-stained section. (20× magnification), panel (**D**) immunohistochemistry section stained using the diagnostic antibody to IDH1-R132H (20× magnification). (**E**,**F**): Preoperative MRI (E, axial fluid attenuated inversion recovery image and F, T1 post-gadolinium axial scan) of a right temporal tumor consistent with Anaplastic Astrocytoma IDH-1 wild-type, grade III (WHO 2016). The red spots indicate the surgical location of the specimen labelled as IDH-WT. (**G**,**H**) Panel G shows Hematoxylin-and eosin-stained section (20× magnification), and panel H shows the immunohistochemistry section stained using the diagnostic antibody to IDH1-R132H that resulted negative (10× magnification). In both cases, direct IDH pyrosequencing confirmed the analysis (codon 132 IDH1 and codon 172 IDH2).

After Raman measurements, the specimens were fixed and provided to the pathologist, blinded to the RS findings, for pathological and molecular analysis (Figure 1) and diagnosis according to the 2016 WHO classification of CNS tumor [1]. As a part of the standard pathological evaluation in our institute, IDH mutational status was assessed using immunohistochemistry and confirmed through direct DNA sequencing. Based on the IDH mutational status, each sample was marked as “IDH-mutated” (IDH-MUT) or “IDH wild-type” (IDH-WT). 

### 2.3. Raman Analysis

A benchtop spectrometer system (model RA800 series-Renishaw plc, Wotton-under-edge, Gloucestershire, UK) was used to collect the Raman spectra from tissue samples. The system ran Renishaw’s WiRE 4.0 software and was equipped with a near-infrared (NIR) laser (785 nm) with a maximum power of 500 mW. The initial spectral region of interest was 90–1800 cm^−1^. We used an exposure time ranging from 0.5 to 2.5 s per spectrum. A range from 1 to 4 spectral accumulations for each acquisition was obtained. The power of the laser was kept at 100% for all the measurements. Spectra were acquired from randomly located points across the sample. The line-focus laser minimized potential photodamage or fluorescence induction.

An automatic performance quality check was performed on silicon and polystyrene internal standards before the beginning of each measurement to reduce sample-to-sample variation. Temperature and humidity conditions, exposure time, laser power, and numbers of accumulations were iteratively optimized for the sample acquisition. A total of 1157 (mean 54.55; SD 6.28; range 35–66) points was used for spectral acquisition for all the samples (a single spectrum for each point). A total of 504 (mean 55.29; SD 5.37; range 46–66) points were analyzed for IDH-WT group. A total of 653 (mean 53.95; SD 7.01; range 35–64) points were analyzed for IDH-WT group.

We obtained the maximum number of spectra with a higher S2N ratio with a punctual acquisition mode for each sample. We completed the assessment on each sample within 60 min from the withdrawal to reduce the biological changes and to best simulate an in-vivo analysis [14].

### 2.4. Data Processing and Classification Models

#### 2.4.1. Data Processing

Pre-preprocessing was performed before model classification to remove the signal’s baseline drifts [15], cosmic rays, and background artefacts [16]. Data pre-processing (Matlab 2019b Mathworks, Natick, MA, USA) followed these steps: (1) truncation of the spectral range to 400–1750 cm^−1^; (2) outlier removal using interquartile filtering; (3) Multiple Scatter Correction (MSC) normalization (4) selection of spectra with Signal-to-Noise (S2N) ratio ≥3.5 around the phenylalanine 1004 cm^−1^ peak (4) background signal subtraction via Vancouver Raman Algorithm (VRA) method [17]; (5) Savitzky-Golay Filter (3rd order, 9 point window); signals normalization via global min–max values.

**Figure 2:**
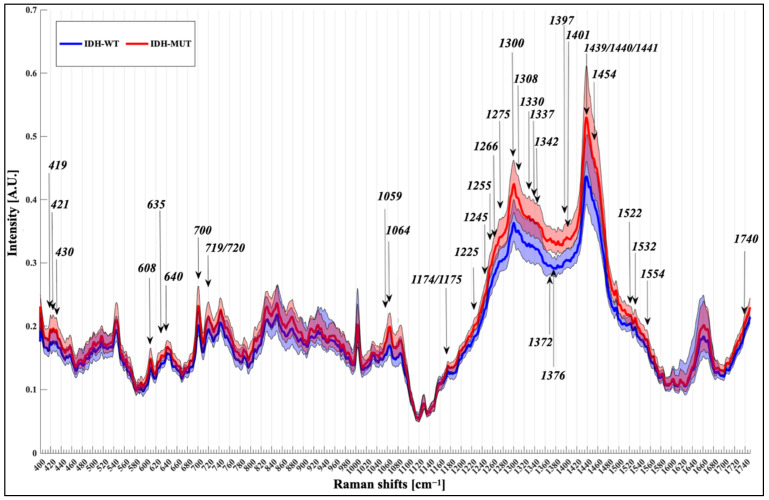
Normalized median spectra with IQR for IDH-WT specimens (blue) and IDH-MUT specimens (red). Arrows mark the most discriminant peaks with a known biological assignment.

The resulting spectra were respectively 653 for IDH-MUT and 504 for IDH-WT. Figure 2 reports the normalized median spectra with the 1st and 3rd interquartile bounds for each group. The frequency of all notable peaks and slopes were outlined on the median Raman chart and were added to those reported in the pre-existing bibliography [9,10]. We detected 103 Raman shifts and we used the intensity at each frequency as input features for the classification algorithms. 

#### 2.4.2. Classification Models

Studies focusing on interpretable Machine Learning models are of particular interest to the field, as they could yield physical insights from automatically highlighted patterns in the data. The Support Vector Machine with Radial Basis Function kernel (RBF-SVM) and the eXtreme Gradient Boosted trees (XGB) are two of the most well-known learning methods due to their theoretical performance guarantees and strong experimental results [18]; we thus decided to investigate these models [19,20,21]. We trained each classifier in Leave-one-patient-out cross-validation (LOPO) to achieve a balanced trade-off between performance and robustness. A further nested 5-fold cross-validation was performed during a hyper-parameters optimization. Feature selection was achieved using a statistical algorithm (ANOVA–ScikitLearn Fclassif), providing the top 52 features with the highest discrimination ability among the two datasets.

Parameters for the hyper-parameters grid search were:

- XGB parameters were set to default except for step size shrinkage (eta) (0.01, 0.05, 0.1, 0.2, 0.3); tree method was set to ‘hist’, the learning objective was set to: binary-logistic; the evaluation metric to: negative log-likelihood (logloss); the maximum depth of a tree: (5, 8, 10, 12, 15), the minimum loss reduction required to make a further partition on a leaf node of the tree (gamma): (0.1, 0.2, 0.3, 0.4).
- Grid search parameters for SVM were set default except for the kernel set to Radial Basis Function (RBF), and the regularization parameter (C):(0.01, 0.1, 1, 10, 100, 1000).

### 2.5. Statistical Analysis

Statistical analysis was conducted on the top 52 Raman shifts supplied by the algorithm to confirm the statistical difference between the two groups at each shift. A Mann–Whitney test (two-tailed, α = 0.05) was performed after checking normality using the Shapiro–Wilk test (software SPSS statistics 25.0; IBM SPSS Inc., Chicago, IL, USA). Mann–Whitney U values and *p*-values were calculated for each top Raman shift and reported. Mann–Whitney *N* value was the same for all the tests calculated: *n*_1_ = 504; *n*_2_ = 653; *n*_1_ + *n*_2_ = *N* = 1157. 

## 3. Results

### 3.1. Classification Performances

We obtained 2073 spectra from 38 un-treated specimens: 1133 marked as IDH-MUT and 940 marked as IDH-WT (Table 1). The resulting spectra obtained after the processing phases were respectively 653 for IDH-MUT and 504 for IDH-WT. From the 103 Raman shifts analyzed as input features by XGB and SVM, the cross-validation loop identified 52 shifts with the best ability in the distinction of the two groups. Table 2 shows the most representative Raman shifts with assigned biological significance.

The RBF-SVM algorithm presented the best average performance in distinguishing IDH-MUT from IDH-WT tumor, with an accuracy of 87%, precision 87%, recall 87% and F1-score 87%. These metrics were calculated on detection outcomes of True Negative (TN), True Positive (TP), False Negative (FN) and False Positive (FP).

RBF-SVM correctly classified IDH-MUT spectra with 90% precision, 87% accuracy, recall 87% and F1-score 87%. RBF-SVM had slightly lower performance in IDH-WT spectral classification with 84% precision, 87% accuracy, recall 87%, and F1-score 85%. The XGB showed an average accuracy and precision of 85% (recall and F1-score: 85%). Average performance metrics from the two classification models are reported in Table 3. 

**Figure 3:**
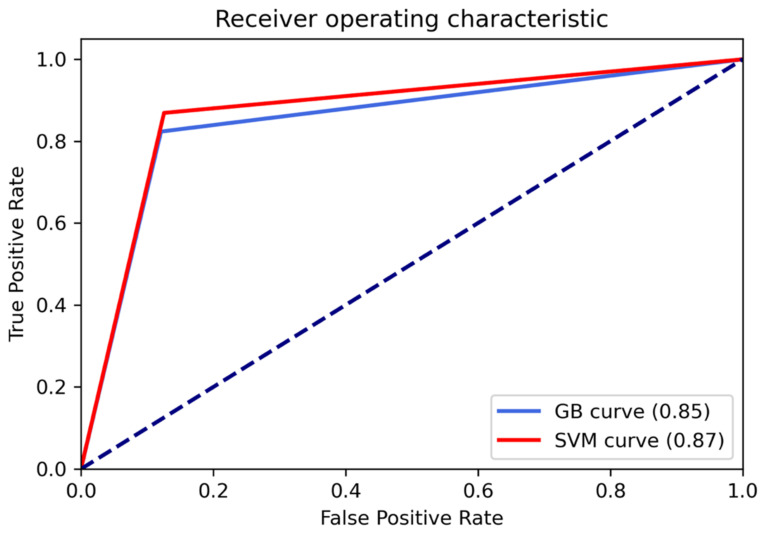
Receiver operating characteristic curve for SVM and XGB.

Receiver Operating Characteristic (ROC) curves were employed to choose algorithms for their performance on the basis of the True Positive Rate (TPR) and False Positive Rate (FPR) and to calculate the Area Under the Curve (AUC). The ROC curves at Figure 3 underlined that RFB-SVM had the best performances in the distinction between the two classes with an AUC of 0.87 compared to 0.85 of XGB. 

### 3.2. Spectral Analysis

A total of 2073 spectra were acquired from 38 different samples: 1133 spectra from 21 samples labelled as IDH-MUT and 940 spectra from 17 specimens labelled as IDH-WT (Table 1). The median spectra of the two groups were plotted and analyzed to identify Raman shifts with possible biological importance. The two algorithms (XGB and SVM) used the intensity of each shift as input features and tested for their discriminative ability. Among 103 peaks adopted as input features, the algorithms identified 52 different Raman peaks with the highest ability in differentiating the two molecular groups. The resulting 52 different spectra were examined to identify shifts with possible known biological significance from the published literature [11,19,20,21,22,23,24,25,26,27,28].

The analysis of the Raman spectra assigned to nucleic acids, proteins, and lipids allowed to identify the different biochemical signature of the IDH-MUT and IDH-WT biopsies (Figure 2).

Both groups were characterized by very intense bands around 1300 and 1440 cm^−1^. These shifts are linked to protein and lipid, and phospholipids. Further notable peaks are evident at band from 600 to 720 cm^−1^, such as peaks at 640 cm^−1^ (cysteine and tyrosin related to protein), 700 cm^−1^ (cholesterol) and 719/720 cm^−1^ (choline; DNA related to nucleic acids group). The group of peaks from 419 to 430 cm^−1^ showed contribution from cholesterol (419/421 cm^−1^) and cholesterol ester (430 cm^−1^). 

All the 52 top peaks identified by the algorithms exhibited statistically significant differences in intensity between the two molecular groups. The prominent regions near 1300 cm^−1^ (*p* < 0.001; U = 74,008), 1439 cm^−1^ (*p* < 0.001; U = 82,982), 1440 cm^−1^ (*p* < 0.001; U = 83,904) and 1441 cm^−1^ (*p* < 0.001; U = 85,905) were reduced in IDH-WT specimens and are linked to CH_2_/CH_3_ deformation of lipids side chains, amino acids, proteins and cholesterol/cholesterol ester [21]. The group of peaks between 1330 cm^−1^ and 1441 cm^−1^ showed the best graphical separation between the two plotted groups, containing 13 out of 52 (25%) of the top peaks analyzed. These shifts are more intense in the IDH-MUT specimens and are related to triglycerides, fatty acids at 1305 cm^−1^ (*p* < 0.001; U = 75,188), proteins, lipids at 1308 cm^−1^ (*p* < 0.001; U = 78,136), 1330 cm^−1^ (*p* < 0.001; U = 85,725), 1337 cm^−1^ (*p* < 0.001; U = 88,005), 1397 cm^−1^ (*p* < 0.001; U = 57,849), 1401 cm^−1^(*p* < 0.001; U = 75,188), 1439 cm^−1^ (*p* < 0.001; U = 82,982), 1440 cm^−1^ (*p* < 0.001; U = 83,904), 1441 cm^−1^(*p* < 0.001; U = 85,095), 1445 cm^−1^ (*p* < 0.001; U = 90,744) and DNA bases at 1342 cm^−1^(*p* < 0.001; U = 90,896), 1372 cm^−1^(*p* < 0.001; U = 43,792) and 1376 cm^−1^ (*p* < 0.001; U = 42,703). Increased content of protein, lipids and phospholipids related to band intensities at 1059 cm^−1^ (*p* < 0.001; U = 100,116), 1064 cm^−1^ (*p* < 0.001; U = 96,524) and 1174–1175 cm^−1^ (*p* < 0.001; U = 91,539 and U = 90,750 respectively) cm^−1^ was also found in the IDH-MUT group. A lower intensity in shifts related to Amide III was observed in IDH-WT tumors at 1225 cm^−1^ (*p* < 0.001; U = 61,672), 1245 cm^−1^ (*p* < 0.001; U = 76,330), 1250 cm^−1^ (*p* < 0.001; U = 71,594), 1265 cm^−1^ (*p* < 0.001; U = 70,384) and 1275 cm^−1^ (*p* < 0.001; U = 66,041). Raman peak related to heme blood is visible at 1454 cm^−1^ (*p* < 0.001; U = 90,818) and is less intense in IDH-WT groups. Intensities of bands related to cholesterol and cholesterol ester sample’s content are seen on the left side of the Raman plot in Figure 2 at 419, 421 and 430 cm^−1^ (more intense in IDH-MUT tumors, *p* < 0.001 and U = 110,116, U = 106,872, U = 108,502 respectively).

## 4. Discussion

In this study we demonstrate that Raman Spectroscopy has the capability to determine the IDH mutational status of fresh glioma biopsies with good precision and accuracy. This can be readily achieved next to the operating theatre and in a short period of time without any additional tissue processing. Our findings added further evidence to the few available studies on molecular characterization of untreated [10] and treated [9] glioma tissue with standard Raman Spectroscopy. Adult patients harbouring IDH mutated low-grade glioma have a better prognosis than IDH-WT tumors after extensive tumor resection [2] and can therefore dedicate, if required, a longer time in rehabilitation after surgery before eventual adjuvant therapy. To date, the presence of IDH mutation is one of the strongest predictors of progression-free survival, overall survival, and response to chemotherapy and radiotherapy. Conversely, IDH WT tumors are often aggressive diseases that must be treated with adjuvant treatment soon after removal. For these reasons, it is crucial to properly balance the extent of resection with postoperative patients’ functional integrity, considering gliomas’ infiltrative nature and the importance of pursuing resection beyond imaging-defined limits. For these reasons, a supratotal resection can be a good strategy in IDH- MUT tumor but would not be the best oncological strategy in the case of IDH-WT tumors, due to the risk of transient neurological impairment that can hamper adjuvant treatments. A rapid, non-invasive, intraoperative technique that can provide an accurate molecular diagnosis is a powerful tool that would allow the surgeon to adapt the surgical strategy to a personalized approach. In the molecular era, the extent of tumor resection has to be critically balanced with the prognostic impact of surgery on different glioma molecular sub-groups and with the risk of postoperative new-onset neurological morbidity due to the closer proximity of the tumor boundaries to formal structures. 

### 4.1. Raman Spectroscopy of Fresh Biopsies Next to the Operative Room

We performed the spectral acquisition within 60 min after surgical samples extraction [14] to avoid any tissue biological or chemical modification. All the analyses were completed adjacent to the theater, and the spectra were obtained from several points of the same specimen. 

Future in-vivo detection of the IDH mutation needs data from fresh samples to replicate an online Raman use and to build a well-structured database that can improve in-vivo Raman analysis. To reach this goal, we decided to use fresh glioma tissue washed only with a saline solution without further treatment. To date, only few studies with data from Raman analysis of fresh and untreated tissue are available [10]. An online tool has been used to characterize molecular tissue changes even in the occurrence of tissue contaminants (i.e., in un-treated specimens) and with a low number of spectra. Data from cryosections or treated specimens can contain, as illustrated before [11,29], different types of artefacts (protein denaturation, cross-linking or loss of lipid) that can interfere with the discrimination process and undermine accurate tissue analysis. After the Raman analysis, the tissue samples were sent immediately to permanent immunohistochemical staining and molecular and pathological analysis that is, to date, the gold standard for diagnosis.

In line with previous results [9,10], this study shows that Raman Spectroscopy can identify chemical differences between IDH-MUT and IDH-WT fresh samples. The chemical characteristics of specimens and some distinctive shifts matched with previous authors’ findings, proving that this technique is reliable and exploitable in different surgical scenarios. Two different algorithms allowed us to evaluate the 52 best representative Raman shifts among the 103 investigated. The RBF-SVM and eXGB showed excellent performances and allowed to distinguish IDH-MUT tumors from IDH-WT tumors with an average accuracy of 87% and 85%, respectively. These results are consistent with the performances reported in previous works on frozen samples [9] (ranging from 88 to 89%) and on fresh tissues (sensitivity and specificity ranging from 91 to 95%). RBF-SVM correctly classified Raman spectra with a precision of 90% in IDH-MUT tumors and 84% in the case of IDH-WT tumors. 

### 4.2. Raman Spectroscopy and IDH Mutation

IDH mutation in CNS surgery is specific for glioma and can be pivotal in those cases where morphological, epidemiological, and radiological factors are confusing. In IDH-1 and IDH-2 mutated tumors, the abnormal production of D-2-HG is related to a profound alteration in cell metabolism, causing modifications in energetic status, altered response to oxidative stress, mutations in DNA and histones methylation status. Furthermore, Koivunen et al. demonstrated that D-2-HG could decrease the production and activity of HIF-1α, with a critical role in the cellular response to hypoxia and angiogenesis. This alteration can increase the risk of DNA damage and mutation due to a rise in cellular oxidative stress [30]. 

Our work demonstrated that the biochemical changes induced by IDH mutation could be detected by the mean of RS and exploited in fresh tissue glioma biopsies to distinguish between IDH-MUT and IDH-WT tumors. This tool, measuring the biological consequence of both IDH1 and IDH2 mutation, can overcome some limitations of immunohistochemistry that is sensitive to the common IDH1 mutation. RS could therefore be an essential tool in the molecular diagnosis of these tumors and minimize the error rate if the surgeon approaches a lesion that harbors a rare IDH mutation.

Furthermore, reliable and accurate information regarding IDH mutational status before or during surgery can help target glioma in surgical scenarios with genotype-specific local treatment against IDH, avoiding systemic toxicity [31]. 

### 4.3. Raman Shifts

From the 103 Raman shifts analyzed, we identified 52 shifts with the highest performance of samples distinction. In particular, these peaks were related to well-known biological components such as proteins (635, 640, 1174–1175, 1225, 1245, 1250, 1275, 1308, 1330, 1401, 1412, 1522, 1554 cm^−1^), nucleic acids (720, 743, 1342, 1372 cm^−1^ and 1376 cm^−1^), lipids (419, 421, 430, 608, 700, 719, 1059, 1064, 1255, 1300–1305, 1337, 1440 cm^−1^), heme groups within the samples (1454 cm^−1^) and carotenoid (1532 cm^−1^). Raman shifts showed higher intensity in IDH -MUT biopsies than IDH-WT at 419 cm^−1^ (cholesterol), 635 cm^−1^ (tyrosin), 1059/1126/1266/1305/1445/1740 cm^−1^ (triglycerides/fatty acids). The same data was confirmed by Livermore et al. [10]. Uckermann et al. [9] in their study on frozen tissue showed, similar to our work, a higher intensity for IDH-MUT glioma in peaks at 640 cm^−1^ (cysteine), 1174 cm^−1^ (proteins), 1337 cm^−1^ (lipids and proteins), suggesting a changed protein profile between the two molecular sub-groups. Furthermore, peaks at 424, 430, 608, 720, 743, 808, 1215, 1225, 1245, 1255, 1275, 1300, 1308, 1330, 1354, 1366, 1376, 1385, 1390, 1397, 1401, 1412, 1439, 1440, 1454, 1495, 1502, 1532, 1554 and 1705 cm^−1^ are for the first time reported in this study compared to the few previous study reporting data about RS and IDH mutation [9,10] in fresh and frozen samples. In particular, peaks at 430, 608, 720, 743, 1225, 1245, 1255, 1275, 1300, 1308, 1330, 1376, 1397, 1401, 1412, 1439, 1440, 1454, 1532, and 1554 cm^−1^ belong to the most discriminant peaks with a known biological assignment as shown in Table 2. In the future, other groups could add these 18 new shifts with attested biological relevance to increase the power of Raman classification methods in-vivo or onto un-processed tissue. 

### 4.4. Study Limitation

The small sample size of specimens analyzed and the time requested for each analysis, higher than some of the previously reported studies [5,10], were primary limitations. The limited study population hampers a stronger and more powerful data analysis. However, this study is one of the few clinical series available and represents a valuable contribution to this methodic. The decrease in the number of spectra before and after the pre-processing and processing steps indicates the need for particular attention to the raw data acquisition process to avoid excessive noise.

Although developed in an actual surgical scenario, the nature of this study was not prospective: the data obtained require validation in a prospective cohort to assess the real impact of this methodic on the surgical decision workflow and patients’ oncological and functional outcomes.

We provided preliminary technical and clinical advances and a variegate spectrum database on untreated tissue to develop further this safe and reliable technology.

## 5. Conclusions

In conclusion, this study demonstrates the ability of Raman spectroscopy to detect changes in the biochemical composition of glioma tumors induced by IDH mutation, ex-vivo onto untreated specimens. The intraoperative detection of IDH mutational status can be of primary importance, allowing the surgeon to tailor the surgical workflow intraoperatively and eventually deliver future in situ therapies. This study adds an important contribution to the available knowledge on this field and is a critical footstep for obtaining an accurate in-vivo intraoperative IDH genotyping.

## Figures and Tables

**Table 1:** Demographic, clinical and pathological characteristics of the study population.

Characteristics	IDH-MUT	IDH-WT
Number of samples	21	17
Number of total spectra acquired (mean)	1133 (53.9)	940 (55.3)
Histological classification (n° of spectra):		
-Astrocytoma, WHO grade II	1 (55)	1 (62)
-Oligodendroglioma, WHO grade II	4 (220)	-
-Astrocytoma, WHO grade III	3 (141)	3 (167)
-Oligodendroglioma, WHO grade III	10(538)	-
-Glioblastoma, WHO grade IV	3 (179)	13 (711)
Mean Age (SD)	42.5 (13.04)	63.23 (9.72)
Sex (%)		
Male	13 (65)	10 (58.8)
Female	7 (35)	7 (41.2)
Median KPS before surgery (range)	100 (70–100)	100 (70–100)
Tumor location (primary lobe involved)		
Frontal	14	7
Temporal	3	6
Parietal	3	3
Occipital	1	1

**Table 2:** Proposed assignment of the main Raman shifts based on pre-existing bibliography [11,19,20,21,22,23,24,25,26,27,28].

Raman Shifts (cm^−1^)	Proposed Assignments
419	Cholesterol
421	Cholesterol
424	Undefined
430	Cholesterol/cholesterol ester
608	Cholesterol
633	Undefined
635	Tyrosine
640	Cysteine, tyrosine
700	Cholesterol
719	Choline in the head group of sphingomyelin and phosphatidylcholine, phosphatidylethanolamine
720	DNA
743	Adenin, DNA, Heme
808	Undefined
1059	Triglycerides/fatty acids
1064	Lipids [C-O stretch and C-O-C symmetric stretch, C-C stretch of phospholipids (side chains specifically) and cholesterol]
1174	Proteins
1175	Proteins
1215	Undefined
1225	Amide III band
1245	Amide III band
1250	Amide III, proteins
1255	Lipids
1265	Amide III band
1266	Lipids
1275	Amide III
1300	Phospholipids, fatty acid, cholesterol
1305	Triglycerides/fatty acids
1308	C-N asymmetric stretching in asymmetric aromatic amines
1330	C-H deformation or CH_2_ bend (proteins)
1337	Lipids and proteins
1342	Nucleic acids
1354	Undefined
1366	Undefined
1372	Nucleic acids
1376	Nucleic acids, DNA
1385	Undefined
1390	Undefined
1397	CH_2_/CH_3_ deformation of lipids and proteins
1401	Protein
1412	amino acids: aspartic & glutamic acid
1439	CH_2_/CH_3_ deformation of lipids side chains, proteins, amino acids, cholesterol/cholesterol ester
1440	CH_2_/CH_3_ deformation of lipids side chains, proteins, amino acids, cholesterol/cholesterol ester
1441	Lipids and Proteins
1445	Lipid
1454	Heme groups
1495	Undefined
1502	Undefined
1522	Proteins
1532	Carotenoid
1554	Tryptophan
1705	Undefined
1740	Lipids

**Table 3:** Performance metrics for RBF-SVM and XGB.

Performance Metrics	RBF-SVM	XGB
Accuracy	0.87	0.85
Precision	0.87	0.85
Recall	0.87	0.85
F1-score	0.87	0.85

## Data Availability

Data acquired for this study are accessible from the corresponding author on reasonable request.
